# Developing a Community-Driven, Locally Sourced Medically Tailored Meal Model: A Pilot Linking Healthcare, Farmers, and Patients

**DOI:** 10.3390/nu18040589

**Published:** 2026-02-11

**Authors:** Julie Brown, Ashton Potter, Leandra Forman, Jairus Rossi, Anna Cason, Christa Mayfield, Alison Gustafson

**Affiliations:** 1 Department of Dietetics and Human Nutrition, College of Agriculture, Food and Environment, University of Kentucky, Lexington, KY 40506, USA; annamcason@uky.edu (A.C.); christa.mayfield@uky.edu (C.M.); alison.gustafson@uky.edu (A.G.); 2 Martin Gatton College of Agriculture, Food and Environment, University of Kentucky, Lexington, KY 40506, USA; ashtonpotter@uky.edu (A.P.); jairusrossi@uky.edu (J.R.); 3 FoodChain, 501 W. 6th Street, Lexington, KY 40508, USA; leandra@foodchainlex.org

**Keywords:** Food is Medicine, medically tailored meals, local food systems, hypertension, type 2 diabetes, food security, farmer economic impact, community capacity building

## Abstract

Background: Food is Medicine (FIM) programs improve health outcomes by integrating nutrition interventions into healthcare delivery, yet gaps remain in community partner capacity and local food system integration. This program aimed to build community capacity to develop a locally sourced, medically tailored meal (MTM) model connecting farmers, healthcare providers, and patients. Methods: Meals were developed by a registered dietitian following American Heart Association (AHA) Heart-Check and MyPlate nutrition standards. Recipes were adapted for freezing, scaled for production, and sourced from 26 central Kentucky farms. Eligible participants with hypertension or type 2 diabetes (T2D) were referred through the Food as Health Program at the UK healthcare screening hub and received 10 meals weekly for 12 weeks. Results: Twenty-five participants enrolled (76% female, mean age 52). Of the 25 that participated, *n* = 12 had complete pre- and post-clinical measures. There was a significant mean decrease in systolic blood pressure from 137.5 to 128.1 mmHg (−9.4 mmHg). There was no significant change in diastolic blood pressure, which went from 79.8 to 70.2 mmHg (−9.2 mmHg), and BMI, which went from 37.59 to 37.93 kg/m^2^ (0.33 BMI). Over 80% of ingredients were locally sourced, generating $10,686 in farm sales. Participant satisfaction and engagement were high. Conclusions: A community-based FIM model using locally sourced, medically tailored meals improved health outcomes and generated measurable economic benefits for local farmers. Strengthening community capacity and integrating such programs into healthcare payment systems are critical next steps for scale and sustainability.

## 1. Introduction

Food is Medicine (FIM) programs encompass a range of strategies for providing healthy food to prevent, manage, or treat chronic conditions in coordination with healthcare systems [1]. Growing evidence links participation in FIM programs to reductions in hospitalizations and emergency department visits [2] and improvements in diet quality, blood pressure, and glycemic control. Specifically, a recent produce prescription analysis found that adults with uncontrolled diabetes experienced a reduction in HbA1c levels by about 0.7 percentage points [3]. Another study found that adults with hypertension, systolic and diastolic blood pressures declined by 8.38 mmHg [4].

However, there are equally robust studies indicating no change in clinical outcomes, such as in HbA1C [5]. While the field of Food is Medicine requires further robust clinical trials, there is growing policy advocacy towards advancing these programs as a covered benefit within certain insurance programs [6]. Thus, while there remains the need to examine the effectiveness of these programs, at the same time communities need to help local partners become viable partners in the FIM space. There remain challenges related to delivery models, duration, and integration with local food systems.

While many FIM programs operate through large-scale partnerships or food pantries, fewer emphasize community-supported agriculture, farmers’ markets, or local food delivery services. Locally produced food purchases can generate significant economic returns, with research showing multiplier effects of $1.32–$1.90 per dollar spent [7]. Despite the economic benefits, community organizations often lack the capacity to produce and distribute MTMs that meet dietary guidelines while supporting local economies.

This program sought to design and pilot an MTM intervention that aligns with the American Heart Association’s dietary recommendations for hypertension, builds farmer engagement, and integrates healthcare and community-based delivery systems, while assessing both health and economic impacts.

## 2. Materials and Methods

Generative artificial intelligence (GenAI) was not used in this paper.

### 2.1. Development of the Medically Tailored Meals Program

A registered dietitian (RD) developed MTMs aligned with the AHA Heart-Check Recipe Certification Program nutrition requirements: ≤500 calories, ≤3.5 g saturated fat, ≤600 mg sodium, and ≤2 tsp added sugar per serving [8]. Meal design incorporated *MyPlate* and the 2020–2025 *U.S. Dietary Guidelines for Americans*, emphasizing whole grains, fruits, vegetables, lean proteins, and low-fat dairy [9,10].

FoodChain (Lexington, KY, USA), a nonprofit with an established community kitchen and expertise in scratch cooking and local food sourcing, served as the production partner for preparing, modifying, and scaling the MTMs. Working with FoodChain staff, the RD selected and adapted AHA recipes suitable for freezing and reheating, considering texture, flavor, and appearance [11]. Recipes were modified for available local ingredients, scaled for 25–125 servings, and analyzed with ESHA Food Processor Software (version 11.15.1, ESHA Research, Salem, OR, USA) to ensure compliance [12]. Nutrition facts labels were generated for each finalized recipe.

### 2.2. Screening and Referral Infrastructure

Participant identification and referral occurred through the Food as Health Program (FAHP) statewide hub [13]. Eligible adults (≥18 years) living in Fayette County, Kentucky, with hypertension or type 2 diabetes who reported food insecurity were referred by healthcare providers.

FAHP staff completed consent and baseline assessments, including demographic data, food security status, height, weight, blood pressure, fasting glucose, and hemoglobin A1c. BMI was automatically calculated in REDCap (version 16.0.9, Vanderbilt University, Nashville, TN, USA) [14,15]. Enrolled participants were then linked to meal delivery coordinated through FoodChain.

### 2.3. Delivery of Medically Tailored Meals

DoorDash, Inc.(San Francisco, CA, USA) partnered with FAHP and FoodChain to manage weekly home deliveries. Referral coordinators entered participant information and delivery instructions into the DoorDash portal. Deliveries occurred on Saturdays between 10 a.m. and 1 p.m., with Monday evening (5–6 p.m.) as a backup.

FAHP coordinators monitored deliveries and resolved logistical issues to ensure consistent access to meals. The partnership maintained food quality and reliability while minimizing missed deliveries.

### 2.4. Farm Impact Measurement Methods

The current figures do not include the costs of overhead, equipment, and packaging, though the latter was estimated as $4 ($0.40 per meal) in the overall per week cost per person. Most organizations who operate this type of program (e.g., incubator kitchens, food hubs) are likely providing a variety of other food preparation, aggregation, and distribution services. The equipment, facility, and infrastructure costs are generally incurred at the time of establishing the organization. Given that FoodChain has been in existence for over 10 years, they already had many of the required resources; therefore, their inclusion in the cost per meal calculation is difficult to disentangle. For the purposes of this analysis, we will not yet include these costs, though could in the future.

Another consideration is that the development of menus and recipes is extremely time-consuming and requires skilled personnel to translate these menus and recipes into a program that delivers healthy meals to participants. These costs are incurred at the outset of program design and so should be considered in the first year or two of a similar program. Once established, however, the skilled labor cost will be reduced each year as institutional knowledge is developed. At this point in the analysis, we are not including this important labor costs because it requires a bit more consideration.

To estimate the proportion of program spending directed toward local agriculture, an 80% local sourcing metric was calculated by assessing the share of ingredient costs attributable to locally sourced items for each recipe. These values were averaged across all meals produced to generate an overall estimate, although most recipes exceeded the 80% threshold. The same approach was used to calculate an average local ingredient cost per person per meal ($3.56), which served as the basis for projecting farm-gate economic impact. Annualized impact estimates were generated by extrapolating the 12-week pilot to a 52-week program operating at 10 meals per person per week, resulting in an estimated $1851 per participant per year. For illustrative purposes, projections were scaled to a cohort of 100 participants, reflecting a conservative and feasible near-term program size, though organizational capacity may support expansion to a larger population.

### 2.5. Key Program Takeaways and Outreach Summary

Participant feedback guided several program refinements. For example, when participants reported that some meals were too heavily seasoned, recipes were adjusted to reduce spice levels and improve overall acceptance. Saturday deliveries were scheduled to support food safety and product quality as most participants were home at that time and could promptly transfer the frozen meals to their freezers upon arrival, reducing the risk of temperature abuse. Alternative delivery times were arranged when necessary.

Communication efforts included a total of 759 text messages, 58 phone calls, and 15 automated behavior-nudge texts across all participants. Of 225 text-based surveys, 116 (52%) received responses. Educational materials promoted local nutrition and chronic disease workshops. Participants consistently reported high satisfaction, appreciating convenience, taste, and the use of local ingredients.

## 3. Results

Participant demographic characteristics are presented in Table 1. Twenty-five participants enrolled (76% female, mean age = 52 years). Most identified as African American (68%) and reported household incomes below $15,000 (60%).

Baseline and post-program clinical outcomes are presented in Table 2. Baseline measures: BMI = 40.8 ± 10.2 kg/m^2^; systolic BP = 136.6 ± 27.4 mmHg; diastolic BP = 79.8 ± 12.1 mmHg. Post-program measures: BMI = 37.5 ± 16.0 kg/m^2^; systolic BP = 131.3 ± 12.0 mmHg; diastolic BP = 73.3 ± 10.0 mmHg. The changes across these clinical outcomes were statistically significant from baseline to post-program.

Over 80% of meal ingredients were locally sourced, averaging $3.56 per meal. The program generated $10,686 in farm sales from 26 central Kentucky farms—approximately $426 per participant or $35.50 per week. Scaled to 100 participants annually, this model could yield ≈ $185,000 in farm-gate revenue.

In a post-program survey we asked participants about their level of satisfaction with the meal delivery program. In general, participants reported strong satisfaction and appreciation for meal quality, convenience, and inclusion of fresh, local ingredients.

## 4. Discussion

This pilot study demonstrates the feasibility, acceptability, and potential impact of implementing a community-driven MTM program designed to integrate local food systems with healthcare delivery. The clinical improvements observed—specifically reductions in both systolic and diastolic blood pressure and modest decreases in BMI—align with existing FIM research showing that nutrition-focused interventions can improve cardiometabolic outcomes among individuals with chronic disease [1,2]. Although previous evaluations have found that BMI changes are often minimal or inconsistently reported [16], our program measured reductions among the 60% of participants with complete pre–post BMI data. This suggests that a structured MTM model emphasizing nutrient-dense, portion-controlled meals may support weight and metabolic improvements when paired with consistent weekly meal access.

Participant feedback further supports the program’s feasibility and high acceptability. More than half of participants engaged in satisfaction polling, with all respondents reporting that they enjoyed the program. Qualitative comments emphasized meal quality, variety, and the convenience of reliable delivery—key components that likely contributed to sustained engagement and reduced barriers to healthy eating. These perspectives reinforce the importance of culturally appropriate, palatable meals in MTM interventions and highlight the value of integrating participant feedback into ongoing program refinement.

In addition to clinical benefits, the program generated meaningful economic contributions to the local agricultural sector. Over 80% of ingredients were sourced from regional farms, providing direct financial benefit to 26 producers. These findings align with national analyses indicating that local food procurement can amplify community-level economic impact and support resilient regional food systems [7]. The economic results also correspond with research showing that value-added agricultural enterprises—including ready-to-eat or minimally processed foods—can strengthen farm income, although operational challenges and production costs may constrain scalability without supportive infrastructure or payment mechanisms [17]. The integration of MTMs into local food value chains may therefore represent an opportunity to expand farm market channels while advancing health equity.

This pilot offers several lessons for practice. Strong community partnerships were essential for coordinating referrals, meal preparation, and delivery logistics. The use of a flexible delivery platform ensured consistent access to meals, while real-time communication between program staff and participants helped maintain engagement and troubleshoot challenges. Iterative recipe adjustments based on participant feedback improved satisfaction and demonstrated the importance of responsiveness in community-based FIM models.

There are several limitations of this feasibility pilot study. This was not a powered study to look at clinical changes and thus no conclusions can be made about clinical relevance. In addition, it was difficult to capture post-program data for clinical outcomes and this limitation needs to be addressed in future studies. This program highlights the need for automatic data transfer between healthcare providers and community partners for improved clinical tracking. Our study had a very small sample size, lack of control group, short follow-up period, and potential measurement variability in blood pressure and weight. This was not a clinical trial and thus we did not capture medication changes or adherence or if participants utilized other intervention programs. Our study was presented to inform others how to improve and integrate community partners in the Food is Medicine space, and we want to highlight the challenges and opportunities for engaging with community partners in this work. There were several challenges including setting up delivery windows between Door Dash and the community partner. We implemented a second pick-up time to overcome this barrier. Since smaller community groups typically cannot afford a delivery mechanism, utilizing an outside vendor was paramount for success and might not be realistic for other groups conducting this work. We did not capture how much of the food was consumed or if food was shared within the household. Thus, a more detailed understanding of who consumed the food and how much is warranted. Lastly, there is room to understand how to scale this operation across other groups in diverse settings. Our work can inform us how to assist community groups with development and clinical partnership, but for a larger scale there needs to be dedicated resources to help smaller groups grow their FIM operations.

Future work should examine the long-term sustainability and cost-effectiveness of locally sourced MTM programs, as well as opportunities to integrate them into value-based care, Medicaid waivers, and other reimbursement frameworks [18]. Recently, Centers for Medicare and Medicaid has solicited input on Food is Medicine programs [19]. In addition, there is legislation to cover the cost of produce prescription programs among veterans [20]. While there is much enthusiasm for these programs, there needs to be support for community organizations to be integrated into the framework of how these programs will operate. Additional research with larger sample sizes and longer follow-up periods is needed to better understand dose–response relationships, program retention factors, and the economic implications for farms and healthcare systems. Building community capacity—including culinary, distribution, and referral infrastructure—will be essential for scaling similar models statewide and nationally [18,19].

Because this analysis focused specifically on farm-gate economic impacts, costs related to labor, recipe development, distribution, and administrative overhead were not included. Informal estimates from FoodChain suggest that these components—excluding packaging and delivery—add approximately $1 per meal per participant. Accurately attributing these costs during the pilot period was challenging as FoodChain leveraged shared labor and overhead across multiple concurrent programs, effectively offsetting some expenses through existing operational capacity.

Overall, this pilot contributes to the growing evidence that community-rooted FIM programs can simultaneously advance chronic disease management, strengthen local food economies, and address social determinants of health. By centering local producers and community partners, MTM interventions such as this one may help create sustainable and equitable regional food and health ecosystems.

## 5. Conclusions

This pilot demonstrates the feasibility and acceptability of implementing a community-driven FIM program that integrates medically tailored meals into healthcare referral pathways while intentionally supporting local food systems. The program successfully established partnerships among healthcare providers, a community kitchen, delivery services, and regional farmers, and showed that high levels of local sourcing can be achieved within a medically tailored meal model.

Although not designed or powered to assess clinical effectiveness, the observed improvements in blood pressure and stability in BMI suggest promising trends that warrant further investigation in larger, controlled studies. Equally important, this pilot highlights the operational considerations, infrastructure needs, and community capacity required to implement and sustain locally sourced MTM programs.

Overall, these findings support the use of community-based MTM initiatives as a feasible and scalable approach within the FIM landscape. Future research should focus on rigorous evaluation of clinical effectiveness, cost-effectiveness, and reimbursement pathways, as well as strategies to support community organizations as essential partners in the expansion of FIM programs.

## Figures and Tables

**Table 1:** Participant demographics in the FoodChain Food is Medicine pilot *n* = 25.

	%or Mean/*n*	SD
age	52 (25)	9.24
gender		
female	76% (19)	
male	24% (6)	
race/ethnicity		
asian	4% (1)	
african american	68% (17)	
white	28% (7)	
hispanic, latinx/e, or spanish origin		
no	96% (24)	
prefer not to answer	4% (1)	
income		
less than $10,000	36% (9)	
$10,000 to $14,000	24% (6)	
$15,000 to $24,000	12% (3)	
$25,000 to $34,000	16% (4)	
$35,000 to $49,000	12% (3)	
employment status		
employed part-time or full-time	44% (11)	
out of work 1yr or more	12% (3)	
retired	4% (1)	
disabled	40% (10)	
highest level of school completed		
9th–12th; no diploma	20% (5)	
high school grad or ged	24% (6)	
vocational trade or business school program	8% (2)	
some college, no degree	28% (7)	
associate degree	8% (2)	
bachelor’s degree	4% (1)	
graduate or professional degree	8% (2)	
household size		
1	28% (7)	
2	44% (11)	
3	20% (5)	
4	0% (0)	
5	4% (1)	
6	4% (1)	
marital status		
married	12% (3)	
not married, living with partner	4% (1)	
never married	56% (14)	
divorced	16% (4)	
separated	12% (3)	
food assistance program received in last 30 days		
snap	23% (8)	
wic	0% (0)	
food pantry	8% (2)	
none	60% (15)	
body mass index (bmi)	40.75 (22)	10.22
blood pressure		
systolic	136.59 (17)	27.42
diastolic	79.82 (17)	12.13

**Table 2:** Pre- and post-program *n* = 25/*n* = 15.

	Pre	Post	Coefficient and 95% CI
blood pressure			
systolic mmhg	137.46	128.06	−9.4 (−14.81, −3.98), *p* = 0.02
diastolic mmhg	79.4	70.2	−9.2 (−26.9, 8.51), *p* = 0.48
body mass index (bmi) (*n* = 15)	37.93	37.59	0.33 (−0.52, 1.21), *p* = 0.87

## Data Availability

The data presented in this study are available on request from the corresponding author (A.G.). The data are not publicly available due to protection of participant identities, and all provided data will be de-identified.

## Abbreviations

The following abbreviations are used in this manuscript:

AHA	American Heart Association
FAHP	Food as Health Program
FIM	Food is Medicine
MTM	Medically tailored meal
